# Miliary opacities in pulmonary sarcoidosis

**DOI:** 10.1002/rcr2.563

**Published:** 2020-04-18

**Authors:** Keishi Sugino, Hirotaka Ono, Masahiro Ando, Seiji Igarashi, Atsuko Kurosaki, Eiyasu Tsuboi

**Affiliations:** ^1^ Department of Respiratory Medicine Tsuboi Hospital Koriyama City Japan; ^2^ Department of Pathology Tsuboi Hospital Koriyama City Japan; ^3^ Department of Diagnostic Radiology Fukujuji Hospital, Japan Anti‐Tuberculosis Association Tokyo Japan

**Keywords:** Miliary opacity, non‐caseating epithelioid cell granuloma, sarcoidosis

## Abstract

Pulmonary sarcoidosis should be considered in the differential diagnosis of miliary opacities in bilateral upper lobes predominance.

## Graphical abstract

We present a rare case of a 37‐year‐old woman diagnosed with pulmonary sarcoidosis who presented with unusual bilateral upper lobes miliary opacities on chest high‐resolution computed tomography.

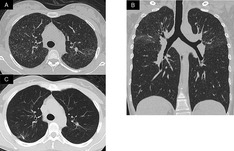

## Clinical Image

**Figure 1:**
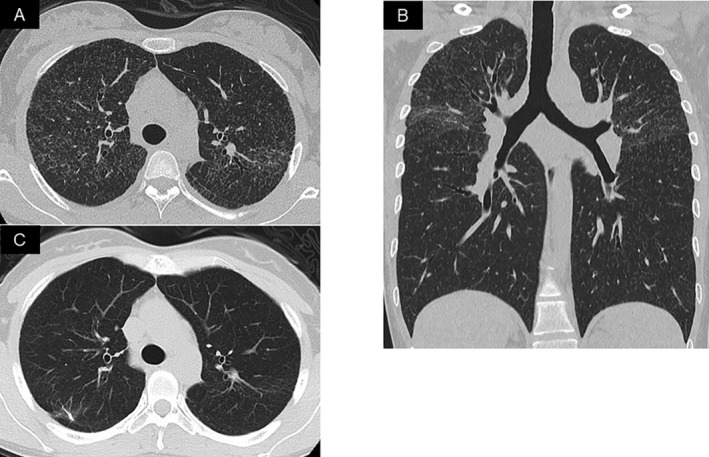
Chest high‐resolution computed tomography (HRCT) revealed miliary opacities in bilateral upper lobes predominance. (A) Transverse section on chest HRCT and (B) coronal images of chest CT. (C) At three months, after corticosteroid therapy, miliary opacities in both lung fields markedly improved.

Typical radiological changes of pulmonary sarcoidosis consist of hilar lymph node enlargement and micronodules along the peribronchovascular bundles; miliary opacities are rare. A previously well 37‐year‐old female presented with a six‐month history of dry cough, fatigue, and weight loss. Serum angiotensin‐converting enzyme (ACE; 27.7 U/L) and soluble interleukin‐2 receptor (sIL‐2R; 674 U/mL) levels were elevated. Chest high‐resolution computed tomography (HRCT) revealed bilateral upper lobes miliary opacities (Fig. 1A, B). 18F‐fluorodeoxyglucose–positron emission tomography revealed mild hypermetabolism in the lung abnormalities. Examination of bronchoalveolar lavage (BAL) fluid revealed a lymphocytosis of 20.0% (normal <14%) and a normal CD4/CD8 ratio of 2.2. Sputum and BAL fluid cultures were negative for fungal, bacterial, and mycobacterial pathogens. Lung biopsy specimens from the right upper lobe obtained by video‐assisted thracoscopic surgery revealed scattered non‐caseating epithelioid cell granulomas in the alveoli and pleura, confirming the diagnosis of pulmonary sarcoidosis. After three months of treatment with oral corticosteroid, her clinical condition and chest imaging abnormalities markedly improved. In addition, serum ACE and sIL‐2R levels decreased to 10.7 U/L and 157 U/mL, respectively (Fig. 1C). The pattern of military opacities is rare in sarcoidosis (<1% of cases) 1 but should be considered in the differential diagnosis, in addition to malignancy, tuberculosis, and pneumoconiosis 2.

### Disclosure Statement

Appropriate written informed consent was obtained for publication of this case report and accompanying images.
